# Assessing quality indicators related to mental health emergency room utilization

**DOI:** 10.1186/s12873-019-0223-8

**Published:** 2019-01-15

**Authors:** Marie-Josée Fleury, Marilyn Fortin, Louis Rochette, Guy Grenier, Christophe Huỳnh, Éric Pelletier, Helen-Maria Vasiliadis

**Affiliations:** 1 Department of Psychiatry, McGill University, Montreal, QC Canada; 2 Douglas Mental Health University Institute Research Centre, 6875 LaSalle Blvd., Montreal, QC H4H 1R3 Canada; 3 Quebec National Institute of Public Health, Quebec, QC Canada; 4 Centre de recherche et d’expertise en dépendance, Montréal, QC Canada; 5 Centre intégré universitaire de santé et de services sociaux du Centre-Sud-de-l’Ile-de-Montréal, Montréal, QC Canada; 6 Département des sciences de la santé communautaires, Université de Sherbrooke, Sherbrooke, QC Canada; 7 Centre de recherche de l’hôpital Charles LeMoyne, Longueuil, QC Canada

**Keywords:** Quality of mental health services, Quality indicators, Access to care, Continuity of care, Appropriateness of care, Emergency room, Mental illness

## Abstract

**Background:**

This descriptive study compared 2014–15 to 2005–06 data on the quality of mental health services (MHS) in relation to emergency room (ER) use to assess the impact of the 2005 Quebec MH reform regarding access, continuity and appropriateness of care for patients with mental illnesses (PMI).

**Methods:**

Data emanated from the Quebec Integrated Chronic Disease Surveillance System (Quebec/Canada). Participants (865,255 for 2014–15; 817,395 for 2005–06) were age 12 or over, with at least one MI, including substance use disorders (SUD), diagnosed during an ER visit, outpatient treatment or hospitalization. Variables included: access (ER use/frequency, hospitalization rates, outpatient consultations preceding an ER visit), care continuity (outpatient consultations following an ER visit/hospitalization, consecutive returns to the ERs), and care appropriateness (high ER use, recurrence of yearly ER visits, length of hospitalization). Frequency distributions were calculated on sex, age and geographic area for ER visits/hospitalizations in 2014–15, and between 2014 and 15 and 2005–06.

**Results:**

PMI accounted for 12 % of the Quebec population in 2014–15 (*n* = 865,255), of whom 39% visited an ER for any reason. Amount and frequency of ER use and number/length of hospitalizations were almost twice as high for PMI versus patients without MI; 17% of PMI were also high/very high ER users and were frequently hospitalized. Among PMI, ER users were also frequent users of outpatient services despite a lack of follow-up appointments after ER visits or hospitalizations. Findings revealed some positive changes over time, such as decreased ER and hospitalization rates; yet overall access, continuity and appropriateness of care, as measured in this study, remained low.

**Conclusions:**

This study demonstrated that the Quebec reform did not produce a substantial impact on ER use or substantially improved care, as hypothesized. Better access and continuity of care should be promoted to reduce the high prevalence of ER use among PMI. Quality improvement in MHS may be realized if ERs are supported by substantial and well-integrated community MH networks.

## Background

Quality improvement in mental health (MH) systems is a priority in most countries [1–3]. One in four individuals will experience a mental illness (MI) in their lifetimes [4]. The prevalence of MI is increasing particularly as populations age, with healthcare costs expected to reach $80 billion in Canada by 2021 [5]. In 2013, 60% of the Canadian MH budget was allocated to hospital inpatient services [6]. MH systems in developed countries (United Kingdom, Australia, Belgium, etc.) have undergone major transformation in recent decades aimed at improving overall performance [7–11], primary care [12, 13], service integration [14, 15], care continuity [16], and in adopting multidisciplinary teams, and recovery best-practices (e.g. care pathways, intensive case management, assertive community treatment) [17–19]. In Canada, the 2005 Quebec MH reform [20] followed international trends [7–11], integrating MH and primary care services, and introducing recovery best-practices for improved healthcare quality [21]. International studies supported these reforms in demonstrating that 43–67% of individuals [22–24], did not consult with healthcare professionals for MI or SUD. The ERs became the preferred destination for individuals facing deteriorating MH conditions [25]. Canadian and American studies estimate that 4–12% of ER visits are for MH reasons [26–28], increasing ER use by 15% or more in the past few years [27, 29]. ER use serves as a barometer for estimating quality in MHS.

Indicators used to assess MHS quality and impact on MH reforms include access, continuity and appropriateness of care [2, 30, 31], with ER attendance as a key indicator of health system access [32, 33]. Canadian and international studies have reported elevated rates of high (more than 4) and very high (more than 12) ER visits/year among PMI, particularly those with co-occurring MI and chronic physical illnesses or SUD [34–36], as compared with other patients [36–41]. PMI also tended toward recurrent ER use over many years [38, 42], depending upon symptom severity, or medication adjustment issues [43]. Fear of stigma may have discouraged disclosure among primary care patients, leading to under-diagnosis, and suboptimal patient management, another factor in ER overuse for MH reasons [43, 44]. Studies found that 8–32% of patients are hospitalized for MH reasons [45–48], identifying a relationship between high ER use and psychiatric hospitalization [49]. Among PMI, high ER users also use outpatient services frequently [37, 49]. Their multiple, serious needs persist despite concerted professional efforts to address them [35]. As PMI receive little attention to their problems in daily living [22–24], the ERs become the first point of service for conditions that do not require emergency services. One US study suggested that treatment in community-level services was adequate for many MH cases, as 20–40% those treated in ERs were not urgent [50]. This situation reflects lack of access to care elsewhere in the MH network [51] and the inability of patients to obtain appointments in a timely manner.

The avoidance of repeated ER visits/hospitalizations through better continuity of care following an ER visit or hospitalization is critical for MHS improvement, especially among patients with more severe and co-occurring conditions [52]. Outpatient appointments held within 7 to 30 days of discharge may address potential side effects associated with medication changes, and promote treatment compliance [30], avoiding return ER visits/hospitalizations, which occur within 30 days after discharge in 10–15% of cases [53]. One study found that only 17% of re-hospitalized patients had received a follow-up appointment prior to discharge [54]. Moreover, PMI remained in hospital longer than others [55–58], increasing the likelihood of readmission as compared with those discharged after brief admissions [55]. Best practices recommend short hospitalizations [55], and close post-discharge follow-up [59] for enhancing social integration and recovery.

Previous studies have assessed the impact of MH reforms [60, 61] in terms of service utilization, access to care, ER (re)admission rates, and hospitalization [62–64]. Others evaluated best practices in patient management [65]. Yet, to the best of our knowledge, no study has focused on quality indicators related to ER use for any reason among PMI in the context of system reforms, including access, continuity or appropriateness of care. Moreover, very few studies have assessed ER use or service quality using large samples of PMI, or included robust longitudinal frameworks [46, 66, 67]. Based on data from the Quebec Integrated Chronic Disease Surveillance System (QICDSS), this descriptive study compared data from 2014 to 15 and 2005–06 on MHS quality indicators in relation to ER use, in order to evaluate the impact of the 2005 Quebec MH reform on access, continuity and appropriateness of care provided to PMI. We hypothesized that overall ER use would be reduced over the course of the reform, and the integration of ER and MH medical services in Quebec health networks reinforced.

## Methods

### Background and data sources

All Canadian residents are covered by universal health insurance, mainly administrated by the provinces and territories [68]. Quebec MHS networks include psychiatric hospitals or psychiatric departments in general hospitals, community health and social service centers (CLSCs), general practitioners (GPs) or psychologists in private practice, addiction centers, and community organizations offering crisis services, self-help groups, and the like. Under the Quebec National Institute of Public Health (INSPQ), the QICDSS collects information on prevalence, incidence, and health service use for the entire Quebec population of 8.2 million [69], through billing files for each medical service performed by physicians in the public health system (e.g. GPs, psychiatrists), who are paid on a fee-for-service basis.

The QICDSS brings together administrative data from the following sources: 1) the health insurance registry (FIPA) containing demographic and geographic records on individuals with valid health insurance numbers (HIN); 2) medical acts compensated by the Quebec Health Insurance Regime (RAMQ) (e.g., ER visits); and 3) the hospitalization databank (MED-ECHO), containing information on hospitalization and discharge. Every Quebec resident registered at RAMQ is assigned a unique identifier, or health insurance number (HIN). The HIN is used to link the various data sources comprising the QICDSS. The linkage probability is 100% between the health insurance registry and the medical acts file, and 99% between the registry and the hospitalization file. We collected data for 2014–15 and for 2005–06. The public health ethics committee of the Douglas MH University Institute and the *Quebec Access to Information Commission* evaluated and approved the study.

### Sample

Data were extracted for individuals 12 years old and over, diagnosed with at least one MI, including SUD, during an ER visit, hospitalization, or outpatient consultation for the fiscal year from April 1st 2014 to March 31st 2015, and for the comparison year, 2005–06. Diagnoses were based on the *International Classification of Diseases, Ninth Revision (ICD-9)* and the *Tenth Revision* of the Med-Echo (*ICD-10*), using the following codes: 290 to 319; F00 to F99; 571.0–571.3; K70.0-K70.04; 535.3; K29.2; 425.5; I42.6; 357.5; G62.1; E24.4, E52, G31.2, G72.1, K70.9, K85.2, K86.0; O35.4 (Table 1). MI included anxio-depressive disorder, personality disorder, schizophrenia, attention deficit disorder with or without hyperactivity (AD/HD), and other (e.g. senile dementia simple form, non-organic psychosis) [66]. SUD included alcohol or drug disorders (abuse, dependence). Different combinations of co-occurring disorders, such as MI/SUD, MI/physical illness (e.g. cancer, diabetes) or SUD/physical illness, were included. Final samples consisted of 865,255 patients with MI representing 12.2% of the Quebec population for 2014–15; and 817,395 PMI representing 12.3% of the Quebec population for 2005–06.

**Table 1:** Mental illness codes according to the International Classification of Diseases, Ninth and Tenth revisions

Diagnoses	*International Classification of Diseases, Ninth Revision (ICD-9)*	*International Classification of Diseases, Tenth Revision* (*ICD-100*
Schizophrenia and psychotic disorders	295, 297, 298	F20, F21, F22, F23, F24, F25, F28, F29, F32.3, F33.3, F44.89
Bipolar disorders	296.0, 296.1, 296.4, 296.5, 296.6, 296.8	F30.1, F30.2, F30.3, F30.4, F30.8, F31.1, F31.6, F31.2, F31.73-F31.78, F31.81, F31.9, F38
Depressive episodes	296.2, 296.3, 296.9, 300.4, 311.9	F32.0, F32.1, F32.2, F32.3, F32.4, F32.5, F32.9, F33.0, F33.1, F33.2, F33.3, F33.9, F33.41, F33.42, F39, F34.8, F34.1, F32.9
Anxiety disorders	300 (except 300.4)	F40-F48, F68
Personality disorders	301	F60, F070, F340, F341, F488, F61
Alcohol use disorders	291, 303.9, 305.0 (alcohol abuse or dependence); 357.5, 425.5, 535.3, 571.0–571.3 (alcohol-induced conditions); 980.0, 980.1, 980,8, 980.9 (alcohol intoxication)	F10.1, F10.2 (alcohol abuse or dependence); F10.3-F10–9, K70.0-K70.4, K70.9, G62.1, I42.6, K29.2, K85.2, K86.0, E24.4, E52, G31.2, G72.1, O35.4 (alcohol-induced conditions); F10.0, T51.0, T51.1, T51.8, T51.9 (alcohol intoxication)
Drug use disorders	292 (drug-induced mental disorder); 304.0–304.9, 305.2–305.7, 305.9 (drug abuse or dependence); 965.0, 965.8, 967.0, 967.6, 967.8, 967.9, 969.4–969.9, 970.8, 982.0, 982.8 (drug intoxication);	F11.1, F12.1, F13.1, F14.1, F15.1, F16.1, F18.1, F19.1, F11.2, F12.2, F13.2, F14.2, F15.2, F16.2, F18.2, F19.2 (drug abuse or dependence); F11.3-F11.9, F12.3-F12.9, F13.3-F13.9, F14.3-F14.9, F15.3-F15.9, F16.3-F16.9, F18.3-F18.9, F19.3-F19.9 (drug-induced mental disorder); F11.0, F12.0, F13.0, F14.0, F15.0, F16.0, F18.0, F19.0, T40, T42.3, T42.4, T42.6, T42.7, T43.5, T43.7-T43.9, T50.9, T52.8, T52.9 (drug intoxication)
Adaptation disorders	308, 309, 311, 313	F43.2; F93.0; F94.0
Attention deficit disorder with or without hyperactivity	314	F90.0
Others mental illnesses	290, 291, 293. 294, 302, 307, 310, 312, 315, 317–319, 571.0–571.3	F00 to F09, F17, F38, F39, F50-F59, F61-F69, F70-F79, F80–89, F90–99

### Variables

Based on the literature [1, 2, 30], the following variables were identified: *Access to care:* 1) ER use and frequency of ER use, 2) hospitalization and length of stay, 3) number of outpatient consultations for any reason during year of study among PMI using the ERs; and 4) consultations among PMI without ER use; 5) outpatient consultations within the week preceding an ER visit for any reason; and 6) outpatient consultations for the year prior to an ER visit for MH reasons. *Continuity of care*: 1–2) outpatient consultations for MH reasons within a week, or month, after an ER visit, or 3–4) hospitalization under the same conditions; 5) repeat ER visits for any reason within 30 days after a first visit to ERs, or after a hospitalization; and 6) hospitalization of patients who re-visited the ERs and reasons for re-visits (e.g. MI, SUD, physical illness, co-occurring MI-SUD). *Appropriateness of care:* 1) high and very high ER users, 2) recurring yearly use of ERs among PMI (3 and 5 recurring years vs 1 year of at least one visit to ERs, from 2005 to 06 to 2015–15), and 3) hospitalization for 30 days or longer for any reason.

Socio-demographic data were collected from the INSPQ databanks for sex (male, female), age (12–17 years, 18–24 years, 25–44 years, 45–64 years, and 65 years or over), geographic area (metropolitan census areas: MCAs: Montreal, urban areas: > 100,000 inhabitants, semi-urban areas: < 100,000, rural areas: < 10,000), on material or social deprivation based on education level (without high school diploma), unemployment, average income, proportions of single-parent families, individuals living alone, and individuals separated, divorced, or widowed. For this study, material or social deprivation was classified in quintiles, with the last quintile representing the most deprived group.

### Data analysis

The hospitalization and medical acts files were analyzed as received, with missing information reported in the study limitations. Regarding the health insurance registry, we checked the eligibility of individuals for public health insurance and whether health insurance cards were valid. However, adjustment was made for young adults (women aged 18 to 25 and men aged 18 to 29) to compensate for delays in the renewal of health insurance cards for this group. Admissibility to the study for this group was based on eligibility criteria alone.

Socio-demographic statistics for patients with/without MIs visiting the ERs were calculated first, and group comparisons made. Frequency distributions for each variable were produced for 2014–15, and for 2014–15 vs. 2005–06. An assessment of trends in MHS use for the ten-year observation period was performed, based on the age-standardized method from the 2014–15 age structure of the population, and relevant findings retained. Frequency distributions on ER use, hospitalization, length of hospitalization, and hospitalization 30 days or longer were also computed for patients without MI. Finally, outpatient consultations for PMI who used ERs were compared with those of PMI who did not use ERs during the study period (2014–15) in order to evaluate overall MHS use. No comparison tests were presented, as all associations were significant for the very large sample size. Confidence intervals were also too narrow to provide substantial information.

## Results

### Socio-demographic portrait of PMI in 2014–15

#### Sample

Of 341,030 PMI who used the ERs in 2014–15, 58% were women, and mean age 51 years; 41% resided in Montreal and 22% in other urban areas, with 41 and 45% in the two lowest quintiles of material and social deprivation respectively (data not shown: DNS). Patients without MI who visited ERs included 52% women, with a mean age of 49 years; 38% resided in Montreal and 25% in rural areas; 39 and 37% were in the two lowest quintiles of material or social deprivation (DNS). Regarding quality indicators, few differences emerged between the sexes, but practices advantaged women slightly over men (e.g. higher use of weekly or yearly consultations prior to ER visits, fewer return ER visits within 30 days; Tables 2 and 4).

**Table 2:** Consultations outside emergency rooms (ERs) among patients with mental illnesses (MI), ER users and patients with MI not ER users for any reason, outpatient consultations for any reason within a week prior to an ER visit, and outpatient consultations for mental health (MH) reasons within a year prior to an ER visit, and a week or a month after an ER visit and hospitalization among patients by sex, age, and residential areas in 2014–15 and 2005–06

	Patients with MI users of ERs						Relative trend (%)^d^	Patients with MI not users of ERs						Relative trend (%)^d^
	2014–15			2005–06				2014–15			2005–06			
	Mean^a^	SD	Median	Mean	SD	Median		Mean	SD	Median	Mean	SD	Median	
Patients with MI	17.75	23.96	10.00	17.04	21.29	11.00	0.01	7.53	9.39	5.00	8.48	9.16	6.00	−11.20
Women	17.64	22.40	11.00	17.40	20.34	12.00		8.03	8.77	6.00	9.07	8.80	7.00	
Men	17.90	25.98	9.00	16.54	22.53	9.00		6.79	10.18	4.00	7.54	9.64	5.00	
12–17 years	7.84	12.04	4.00	7.81	11.68	5.00		3.99	5.71	3.00	4.74	6.10	3.00	
18–24 years	9.07	15.27	5.00	9.48	12.93	6.00		5.18	7.80	3.00	6.28	7.06	5.00	
25–44 years	11.86	17.02	7.00	11.89	14.34	8.00		6.83	8.54	5.00	7.38	7.67	6.00	
45–64 years	16.70	22.50	10.00	16.80	20.04	11.00		7.78	8.91	6.00	8.74	8.79	7.00	
65 years+	28.30	29.74	19.00	28.48	28.37	20.00		10.58	12.15	8.00	11.99	12.53	9.00	
Montreal	20.50	26.92	12.00	19.32	23.29	13.00		7.98	9.76	6.00	9.00	9.41	7.00	
Areas> 100,000	16.66	22.45	10.00	16.07	20.05	10.00		7.22	9.55	5.00	8.29	9.82	6.00	
Semi-urban	16.24	22.13	9.00	15.69	20.28	10.00		7.27	9.62	5.00	7.63	8.09	6.00	
Rural	14.63	19.71	8.00	14.42	18.37	9.00		6.76	7.63	5.00	7.57	7.92	6.00	
		Outpatient consultations for any reason within a week prior to an ER visit (yes)^b^					Relative trend (%)^d^	Outpatient consultations for MH reasons within a year prior to an ER visit (yes)^c^				Relative trend (%)^d^		
		2014–15		2005-06				2014–15		2005–06				
		N (ind.)	% (ind.)	N (ind.)	% (ind.)			N (ind.)	% (ind.)	N (ind.)	% (ind.)			
Patients with MI		126,391 (341,037)	37.06	120,070 (326,572)	36.77		0.80	56,079 (124,863)	44.91	42,464 (117,120)	36,26	23.87		
Women		76,149 (198,688)	38.33	73,062 (188,867)	38.68			30,262 (65,193)	46.50	22,449 (60,711)	36.98			
Men		50,242 (142,349)	35.29	47,008 (137,705)	34.14			25,717 (59,570)	43.17	20,015 (56,949)	35.48			
12–17 years		4095 (19,533)	20.96	3220 (14,639)	22.00			3695 (7103)	52.02	2415 (6048)	39.93			
18–24 years		7729 (30,282)	25.52	7525 (26,654)	28.23			6505 (15,333)	42.42	4692 (13,889)	33.78			
25–44 years		27,917 (89,498)	31.19	33,952 (103,693)	32.74			18,629 (36,594)	50.91	16,918 (41,270)	41.02			
45–64 years		36,612 (98,689)	37.10	38,876 (103,170)	37.68			16,055 (32,749)	49.02	13,078 (32,535)	40.20			
65 years+		50,038 (103,035)	48.56	36,497 (78,416)	46.54			11,195 (33,084)	33.83	5361 (23,405)	22.91			
Montreal		58,081 (140,507)	41.34	57,077 (137,893)	41.39			23,952 (55,836)	42.90	19,067 (51,986)	36.68			
Areas> 100,000		27,169 (74,591)	36.42	24,125 (68,187)	35.38			12,610 (26,261)	48.02	8395 (22,197)	37.82			
Semi-Urban		16,300 (50,689)	32.16	15,619 (49,101)	31.81			8202 (17,172)	47.76	6505 (17,441)	37.30			
Rural		24,303 (73,730)	32.96	22,658 (69,690)	32.51			11,021 (24,963)	44.14	8128 (24,728)	32.90			
		Outpatient consultations for MH reasons 1 week after an ER visit					Relative trend (%)^d^	Outpatient consultations for MH reasons 1 month after an ER visit				Relative trend (%)^d^		
		2014–15		2005-06				2014–15		2005–06				
		N (ind.)	% (ind.)	N (ind.)	% (ind.)			N (ind.)	% (ind.)	N (ind.)	% (ind.)			
After an ER visit														
Patients with MI		12,788 (121,378)	10.54	14,842 (115,702)	12.83		−18.26	30,561 (121,378)	25.18	33,938 (115,702)	29.33	−14,16		
Women		6790 (63,367)	10.63	7919 (60,362)	13.12			16,537 (63,867)	25.89	18,117 (60,362)	30.01			
Men		5998 (57,511)	10.43	6923 (55,340)	12.51			14,024 (57,511)	24.38	15,821 (55,340)	28,59			
12–17 years		1021 (7120)	14.34	879 (6015)	14.61			2187 (7120)	30.72	1680 (6015)	27.93			
18–24 years		1595 (14,888)	10.71	1651 (13,738)	12.02			3620 (14,888)	24.32	3681 (30,225)	26.79			
25–44 years		4673 (35,942)	13.00	6530 (41,236)	15.84			11,017 (35,942)	30.65	14,692 (41,236)	35.63			
45–64 years		3742 (31,547)	11.86	4438 (31,893)	13.92			9000 (31,547)	28.53	10,482 (31,893)	32.87			
65 years+		1757 (31,881)	5.51	1344 (22,820)	5.89			4737 (31,881)	14.86	3403 (22,820)	14.91			
Montreal		6188 (53,941)	11.47	6864 (51,618)	13.30			13,525 (53,941)	25.07	14,892 (51,618)	28.85			
Areas> 100,000		2628 (25,528)	10.29	3201 (21,873)	14.63			6590 (25,528)	25.81	6957 (21,873)	31.81			
Semi-Urban		1722 (16,938)	10.17	2039 (17,234)	11.83			4424 (16,938)	26.12	5213 (17,234)	30.25			
Rural		2168 (24,339)	8.91	2646 (24,202)	10.93			5855 (24339)	24,06	6671 (24,202)	27.56			
		Outpatient consultations for MH reasons 1 week after an hospitalization					Relative trend (%)^d^	Outpatient consultations for MH reasons 1 month after an hospitalization				Relative trend (%)^d^		
		2014–15		2005-06				2014–15		2005–06				
		N (ind.)	% (ind.)	N (ind.)	% (ind.)			N (ind.)	% (ind.)	N (ind.)	% (ind.)			
Patients with MI		9531 (135,919)	7.01	6281 (126,668)	4.96		41.42	25,833 (135,919)	19.01	18,164 (126,668)	14.34	32.54		
Women		5294 (76,407)	6.93	3461 (71,576)	4,84			14,670 (76,407)	19.20	9901 (71,576)	13.83			
Men		4237 (59,512)	7.12	2820 (55,092)	5.12			11,163 (59,512)	18.76	8263 (55,092)	15.00			
12–17 years		675 (4135)	16.32	512 (3412)	15.01			1417 (4135)	34.27	1071 (3412)	31.39			
18–24 years		826 (6698)	12.33	693 (6730)	10.30			1867 (6698)	27.87	1782 (6730)	26.48			
25–44 years		2748 (25,277)	10.87	2153 (28,879)	7.46			6961 (25,277)	27.54	5980 (28,879)	20.71			
45–64 years		2628 (34,313)	7.66	1860 (36,360)	5.12			7486 (34,313)	21.82	5913 (36,360)	16.26			
65 years+		2654 (65,496)	4.05	1063 (51,287)	2.07			8102 (65,496)	12.37	3418 (51,287)	6.66			
Montreal		4325 (59,660)	7.25	2698 (56,259)	4.80			10,663 (59,660)	17.87	7629 (56,259)	13.56			
Areas> 100,000		1959 (27,990)	7.00	1366 (24,533)	5.57			5710 (27,990)	20.40	3826 (24,533)	15.60			
Semi-Urban		1466 (20,206)	7.26	937 (18,710)	5.01			4157 (20,206)	20.57	2864 (18,710)	15.31			
Rural		1711 (27,325)	6.26	1222 (26,218)	4.66			5138 (27,325)	18.80	3689 (26,218)	14.07			

^a^Mean number of outpatient visits per year

^b^All types of consultation, i.e. any reason

^c^Consultations for MH reasons only

^d^Relative trends calculated as: last value (in 2014–15) minus initial value (in 2005–06) divided by the initial value (in 2005–06) multiplied by 100 to obtain value in percentage. Age: standardized data

PMI aged 12–17 most frequently received outpatient consultations for MH reasons for the one-year period preceding an ER visit (52%) versus other age groups, as well as for weekly and monthly outpatient consultations following an ER visit (14%; 31% respectively) or following hospitalization for MH reasons (16%; 34%: Table 2). The 12–17 year group rated lowest on most other indicators. The 65+ age group sought most outpatient consultations for any reason in the week prior to an ER visit (49%); but least outpatient consultations in the year prior to an ER visit (34%), and outpatient consultations for MH reasons within a week (6%) or month (15%) following an ER visit or hospitalization (Table 2). The 65+ age group had the highest percentage of return ER visits within 30 days (42%; Table 4), recurrent ER visits over 3 years (6%; Table 5), and hospitalizations exceeding 30 days (9%; Table 6). The 45–64 age group, followed closely by the 25–44 age group, also made the greatest number of ER visits over five consecutive years (5%; Table 5).

#### Differences in ER use by geographic area

Montreal PMI had the most frequent outpatient consultations over a one-year period (mean 21 for PMI using ERs; mean 8 for PMI not using ERs), and for the week preceding an ER visit (41%; Table 2), with the highest rates for: return to ERs within 30 days (34%), re-hospitalization (33%) (Table 4), and hospitalization exceeding 30 days (4%; Table 6). Montreal also had the highest percentage of outpatient consultations for MH reasons in the week following an ER visit (11%; Table 2). Outpatient consultation rates for MH reasons in the year preceding an ER visit were highest in areas with 100,000+ inhabitants (26%; Table 2), as were return ER visits over five consecutive years (4%; Table 5). By contrast, rural areas had the lowest outpatient consultation rates over a one-year period (mean 15 for PMI using ERs; mean 7 for PMI not using ERs), and the lowest rates for the week or month following an ER visit (9%; 24%), or the week following hospitalization (6%; Table 2). Rural areas had the highest rate for three-year recurring ER visits (6%; Table 5), but lowest hospitalization rates exceeding 30 days (2%; Table 6).

### Access to care (2014–15 versus 2005–06)

Nearly 40% of PMI visited ERs in 2014–15, 2.3 visits each, on average; 34% of them were hospitalized for an average 16 days. Between 2005-06 and 2014–15, ER use and hospitalizations decreased by over 3% among PMI (Table 3). By contrast, 22% of patients without MI visited ERs twice a year on average; 20% were hospitalized for an average eight-day stay. ER use and hospitalization decreased by 6 and 2%, respectively, following implementation of the reform (DNS).

**Table 3:** Utilization of emergency rooms (ERs) among patients 12 years old and over with mental illnesses (MI), and among high and very high ER users, and length of hospitalizations in 2014–15 and in 2005–06 for any reason

ER Utilization	2014–15 (N = 865,255)			2005–06 (*N* = 817,395)			Relative trend (%)^e^
	N (ind.)	% (ind.)	Freq. mean^d^	N (ind.)	% (ind.)	Freq. mean	
Patients with MI	341,030	39.41	2.25	326,570	39.95	2.25	−3.45
High users with MI^a^	55,129	16.17	6.01	51,466	15.76	6.09	−0.26
Very high users with MI^b^	2759	0.81	16.72	2817	0.86	17.01	−3.53
Hospitalization	N (ind)^c^	% (ind. Hosp.)	Length hosp.	N (ind)^c^	% (ind. Hosp.)	Length hosp.	Relative trend (%)^e,f^
Patients with MI	116,675	34.21	16.01	108,915	33.35	16.62	−3.32
High users with MI	31,955	57.96	11.85	26,984	52.43	11.95	
Very high users with MI	2001	72.53	7.90	1843	65.42	7.97	

Note. Relative trend in ER use between 2005 and 06 and 2014–15 among patients without MI = −6.47%. Relative trend in hospitalization between 2005 and 06 and 2014–15 among patients without MI = −1.59%. Age: standardized data

^a^Four to 11 ER visits per year (of total 341,030 patients who visited ERs)

^b^Twelve visits or more ER visits per year (of total 341,030 patients who visited ERs)

^c^Number of individuals hospitalized out of 341,030 patients with MI who visited ERs; number of high users or very high users, of total 55,129 high and 2, 759 very high users respectively in 2014–15

^d^Mean frequency calculated per individual who made at least one ER visit

^e^Relative trends calculated as: last value (in 2014–15) minus initial value (in 2005–06) divided by the initial value (in 2005–06) multiplied by 100 to obtain value in percentage. Age: standardized data

^f^Data not available for high and very high ER users

The 341,030 PMI who used ERs in 2014–15 had an average of 18 outpatient consultations (median: 10), compared with 8 (median: 5) among PMI who did not use ERs. Outpatient consultations among ER patients remained relatively stable after 2005–06 (increased of 0.01%), whereas consultations decreased by 11% among PMI who did not use the ERs. More than 37% of PMI had an outpatient consultation within 1 week preceding an ER visit for any reason, and 45% an outpatient consultation within the year preceding an ER visit for MH reasons. From 2005 to 06 to 2014–15, consultations in the week prior to an ER visit increased less than 1%, but increased nearly 24% for the year prior to an ER visit (Table 2).

### Continuity of care (2014–15 vs 2005–06)

Nearly 11% of PMI had an outpatient consultation for MH reasons within the week following an ER visit, and within 1 month for 25% of patients in 2014–15. By 1 week post-hospitalization, 7% of PMI had received an outpatient consultation, whereas 19% received an outpatient consultation for MH reasons within 30 days. From 2005 to 06 to 2014–15, an 18% decrease in outpatient consultations within a week, and 14% within a month following ER visits were observed, but outpatient consultations within a week or a month following hospitalization increased 41 and 33% respectively (Table 2). Among PMI who visited the ERs in 2014–15, 34% returned after discharge within fewer than 30 days, of whom 30% were hospitalized. The principal reason for return to the ERs, whether after an ER visit, or hospitalization, was physical illness (75% ERs, hospitalization), followed by MH reasons exclusively (13%; 15%), then for co-occurring MI/physical illness (7%; 4%). From 2005–06 to 2014–15, an increase of 3% was observed in return ER visits within 30 days (Table 4).

**Table 4:** Emergency room (ER) return visits within less than 30 days and hospitalization rates for any reason among patients with mental illnesses (MI) who re-visited the ERs by sex, age, residential areas and reasons for return visit in 2014–15 and in 2005–06

	2014–15					2005–06					
	N ind.	% ind.	N visits	% visits	% hosp. re-visit	N ind.	% ind.	N visits	% visits	% hosp. re-visit	Relative trend (%)^d^
Patients with MI	114,579 (341,030)	33.60	244,511	31.58	30.29	104,479 (326,570)	31.99	226,666	30.82	25.71	2.92
Women	65,401 (197,727)	32.91	135,581	30.42	28.80	58,614 (188,895)	31.03	123,205	29.51	25.02	
Men	49,178 (142,297)	34.56	108,930	33.15	32.15	45,865 (137,691)	33.31	103,461	32.54	26.52	
12–17 years	4062 (19,529)	20.80	6794	19.70	16.56	3233 (14,642)	22.08	5464	20.33	16.95	
18–24 years	9079 (30,283)	29.98	19,324	28.85	17.11	8263 (26,655)	31.00	17,918	29.10	14.86	
25–44 years	27,260 (89,494)	30.46	59,703	30.47	17.95	31,319 (103,705)	30.20	70,724	30.54	14.88	
45–64 years	31,342 (98,684)	31.76	69,334	31.81	26.64	31,380 (103,156)	30.42	70,126	30.93	22.36	
65 years+	42,836 (103,045)	41.57	89,356	34.52	45.28	30,284 (78,415)	38.62	62,434	33.08	45.60	
Montreal	48,313 (140,567)	34.37	105,516	33.40	35.48	42,185 (137,545)	30.67	90,230	30.75	31.56	
Areas> 100,000	24,572 (74,213)	33.11	51,051	30.58	26.75	21,284 (67,870)	31.36	44,996	30.09	23.01	
Semi-Urban	15,967 (50,689)	31.50	32,827	29.01	28.51	15,778 (48,652)	32.43	33,417	29,52	22.54	
Rural	25,106 (73,754)	34.04	53,679	30.84	24.46	24,237 (69.587)	34.83	55,888	32.37	20.30	
Reasons for return visit											
PI^a^			183,020 (244,511)	74.85				170,401 (226,666)	75.18		
MI^b^			32,134 (244,511)	13.14				34,514 (226,666)	15.23		
MI-PI			16,715 (244,511)	6.84				10,182 (226,666)	4.49		
MI-SUD^c^			5746 (244,511)	2.35				6496 (226,666)	2.87		
MI-SUD-PI			4436 (244,511)	1.81				3431 (226,666)	1.51		
SUD			1141 (244,511)	0.47				911 (226,666)	0.40		
SUD-PI			1319 (244,511)	0.54				731 (226,666)	0.32		

^a^Physical illness

^b^Mental illnesses (MI)

^c^Substance use disorders (SUD)

^d^Relative trends calculated as: last value (in 2014–15) minus initial value (in 2005–06) divided by the initial value (in 2005–06) multiplied by 100 to obtain value in percentage. Age: standardized data

### Appropriateness of care (2014–15 vs 2005–06)

Over 16% of PMI who visited ERs in 2014–15 were high ER users (mean 6 visits/year), of whom 58% were hospitalized. Less than 1% were very high ER users (average 17 visits/year), yet 73% of them were hospitalized. From 2005–06 to 2014–15, ER use by high or very high users decreased by 0.3 and 4% respectively (Table 3). Most PMI (72%) presented at an ER for only a single year throughout the 2005–06/2014–15 period; 6% presented over three consecutive years (e.g. from 2012 to 13 to 2014–15); and 4% over five consecutive years (e.g. 2010–11 to 2014–15). From 2005–06 to 2014–15, use of ERs for 3 consecutive years increased by 4%, but for 5 consecutive years decreased by 13% (Table 5). More than 3% of PMI were hospitalized longer than 30 days (Table 6), as compared with 0.3% for patients without MIs (DNS). Comparing 2014–15 to 2005–06, hospitalizations exceeding 30 days decreased 4% among PMI (Table 6), but 25% among patients without MIs (DNS).

**Table 5:** Recurring emergency room (ER) patients for any reason with mental illnesses (MI) by sex, age and residential areas in 2014–15 and in 2005–06

	2014–15						2005–06						Relative trend (%) 1 year^a^	Relative trend (%) 3 years^a^	Relative trend (%) 5 years^a^
	1 year		3 years		5 years		1 year		3 years		5 years				
	N (ind.)	% (ind.)	N (ind.)	% (ind.)	N (ind.)	% (ind.)	N (ind.)	% (ind.)	N (ind.)	% (ind.)	N (ind.)	% (ind.)			
Patients with MI	244,666 (341,030)	71.74	19,479 (341,030)	5.71	12,034 (341,030)	3.53	236,697 (326,570)	72.48	17,905 (326,570)	5.48	13,237 (326,570)	4.05	1.02	4.20	−12.84
Women	141,202 (198,708)	71.06	11,705 (198,708)	5.89	7208 (198,708)	3.63	135,492 (188,865)	71.74	10,570 (188,865)	5.60	8254 (188,865)	4.37			
Men	103,464 (142,316)	72.70	7774 (142,316)	5.46	4826 (142,316)	3.39	101,205 (137,713)	73.49	7335 (137,713)	5.33	4983 (137,713)	3.62			
12–17 years	15,218 (19,533)	77.91	844 (19,533)	4.32	118 (19,533)	0.60	12,024 (14,638)	82.14	487 (14,638)	3.33	56 (14,638)	0.38			
18–24 years	22,060 (30,281)	72.85	1746 (30,281)	5.77	825 (30,281)	2.72	20,102 (26,653)	75.42	1308 (26,653)	4.91	643 (26,653)	2.41			
25–44 years	63,503 (89,491)	70.96	5198 (89,491)	5.81	3904 (89,491)	4.36	73,635 (103,697)	71.01	5950 (103,697)	5.74	5121 (103,697)	4.94			
45–64 years	70,291 (98,682)	71.23	5593 (98,682)	5.67	4504 (98,682)	4.56	73,301 (103,168)	71.05	5949 (103,168)	5.77	5441 (103,168)	5.27			
65 years+	73,594 (103,044)	71.42	6098 (103,044)	5.92	2683 (103,044)	2.60	57,635 (78,415)	73.50	4211 (78,415)	5.37	1976 (78,415)	2.52			
Montreal	103,606 (140,578)	73.70	7420 (140,578)	5.28	4224 (140,578)	3.00	102,162 (137,536)	74.28	7018 (137,536)	5.10	4843 (137,536)	3.52			
Areas> 100,000	52,228 (74,219)	70.37	4412 (74,219)	5.94	3023 (74,219)	4.07	48,534 (67,861)	71.52	3890 (67,861)	5.73	2967 (67,861)	4.37			
Semi-Urban	35,433 (50,684)	69.91	3012 (50,684)	5.94	2046 (50,684)	4.04	34,161 (48,662)	70.20	2854 (48,662)	5.87	2395 (48,662)	4.92			
Rural	52,216 (73,762)	70.79	4497 (73,762)	6.10	2648 (73,762)	3.59	49,780 (69,583)	71.54	3960 (69,583)	5.69	2871 (69,583)	4.13			

^a^Relative trends calculated as: last value (in 2014–15) minus initial value (in 2005–06) divided by the initial value (in 2005–06) multiplied by 100 to obtain value in percentage. Age: standardized data

**Table 6:** Hospitalization for any reason exceeding 30 days among patients with mental illnesses (MI) by sex, age, and residential areas in 2014–15 and 2005–06

	2014–15		2005–06		Relative trend (%)^a^
	N (ind.)	% (ind.)	N (ind.)	% (ind.)	
Patients with MI	27,533 (865,255)	3.18	24,326 (817,395)	2.98	−4.28
Women	14,876 (509,452)	2.92	13,566 (489,747)	2.77	
Men	12,657 (355,534)	3.56	10,760 (328,049)	3.28	
12–17 years	480 (64,865)	0.74	434 (39,455)	1.10	
18–24 years	840 (67,742)	1.24	984 (53,189)	1.85	
25–44 years	2753 (239,391)	1.15	3044 (267,018)	1.14	
45–64 years	5162 (288,380)	1.79	5193 (300,173)	1.73	
65 years+	18,298 (203,537)	8.99	14,674 (158,296)	9.27	
Montreal	14,157 (402,188)	3.52	12,162 (394,870)	3.08	
Aeras> 100,000	5893 (181,883)	3.24	4866 (167,793)	2.90	
Semi-Urbain	3351 (115,952)	2.89	3090 (100,980)	3.06	
Rural	3981 (161,829)	2.46	3917 (145,704)	2.67	

^a^Relative trends calculated as: last value (in 2014–15) less initial value (in 2005–06) divided by the initial value (in 2005–06) multiply by 100 to obtain value in percentage. Age: standardized data

## Discussion

This study evaluated changes in MHS quality related to ER use in terms of access, continuity and appropriateness of care for a cohort of PMI, comparing data from 2014–15 vs. 2005–06 when the Quebec MH reform was introduced. We hypothesized that overall ER use would decrease, and that service integration would improve between Quebec ERs and other MH medical services. As hypothesized, ERs and hospitalization for any reasons decreased for PMI from 2005–06 to 2014–15; but their decrease in ER use was two times less than that of patients without MI. Outpatient consultations for MH reasons in the year preceding ER visits also improved considerably: in 2014–15, less than half of PMI had no outpatient consultations for the year prior to their ER visit. Contrary to expectation, indicators of continuity of care or appropriateness revealed either deterioration or only slight improvement from 2005–06 to 2014–15. Positive changes were generally less pronounced for PMI compared with patients without MI. However, some improvement occurred on outpatient consultations for MH reasons following hospitalizations; they increased considerably but were very low overall. Regarding ER visits over 5 consecutive years, rates decreased slightly over time.

The overall findings confirmed a 12% annual prevalence of PMI, similar to rates reported in other North American epidemiological studies, ranging from 12 to 18% [70, 71]. Concerning ER use among PMI, rates in this study, at close to 40%, were much higher than in other North American studies, at 4–12% [26–28, 46]. Our results may be explained by the fact that we investigated ER use for any reason among a cohort of patients diagnosed with MI over a 12-month period. Other studies have tended to investigate ER use for MH reasons only. Yet our data were less likely to underestimate ER use by PMI, as opposed to studies that reported data on ER use for MH reasons exclusively. Such methodological distinctions limit the usefulness of comparisons.

Regarding socio-demographic composition of the cohort, we noted that PMI were slightly more materially and socially deprived than other ER patients, suggesting considerable vulnerability among PMI using ERs, as portrayed elsewhere [72, 73]. Older patients received lower quality care overall, including fewer outpatient consultations prior to ER visits, and fewer within 1 week or month after an ER visit or hospitalization for MH reasons; their rates of return to ERs within 30 days, hospitalizations exceeding 30 days, and recurrent ER use over 3 years for any reason were also lowest. Under-diagnosis of MI [74, 75] or lower prioritization of MH issues among older patients may explain these lower outpatient consultation rates. The fact that older PMI often presented with chronic, co-occurring physical illness may explain their other poor outcomes, and need for additional care, which drove their return ER visits [76], and extended hospitalization rates further exacerbated by placement issues [56]. By contrast, the 12–17 year group received significantly more MHS before and after ER visits or hospitalizations, aimed at countering MI in adulthood [77, 78]. The MH reform may have played a role in prioritizing youth MH; early-onset MI occurs frequently [79], and carries a poor prognosis [80]. However, younger patients used fewer MHS overall, as highlighted in previous research [81, 82]. In Montreal, higher rates of outpatient consultations, return ER visits within 30 days, re-hospitalizations, and hospitalizations exceeding 30 days may have been due to greater numbers of vulnerable patients with complex problems [83]. More availability of primary care, walk-in clinics without follow-up in Montreal may also have increased outpatient consultations in the week preceding ER visits. Furthermore, rates of outpatient consultations, outpatient consultations following ER visits (within a week, or month) and hospitalizations (within a week), as well as higher recurring ER use over 3 years in rural areas, reflected their relative lack of primary care coverage as compared with cities [72].

Concerning access to services, we observed that ER use, frequency of use, and number/ length of hospitalizations were almost double for PMI versus other patients. International studies confirm the frequency of ER use [34, 37, 49], with hospitalization rates similar to ours at 15–32% [48, 56], and extended hospitalization among PMI [84]. Limited or delayed access to primary care and other services, and inadequate follow-up for PMI may explain these results [10, 50]. Yet decreased ER use and fewer hospitalizations over time did suggest some improvement in access to care. Measures introduced over the course of the Quebec MH reform aimed at reducing ER use/hospitalization and supporting PMI included: increased access to primary care, prevention strategies by MH teams in CLSCs, and increased mobile crisis services, and GPs working with respondent-psychiatrists (shared care). Incentives were also provided to improve MH supports in medical clinics [10, 21]. Decreased ER use and hospitalization rates in this study were contrary to results obtained in other North American studies, but only those for MH reasons [48, 85].

Overall, PMI who used ERs also used outpatient services twice as often as those who did not use ERs, but patterns of service use remained stable. By contrast, outpatient service use by PMI who did not use ERs decreased by 11%, confirming their high, and inappropriate use of health services [34, 86]. Yet the results also revealed that some patients received intensive, quality outpatient follow-up in terms of monthly access to family physicians. Moreover, close to 40% of patients received outpatient consultations for any reason within a week prior to ER visits, suggesting the occurrence of ER visits on physician recommendation. Yet the fact that most did not consult a physician before ER visits suggested ongoing difficulties with rapid access to medical clinics or psychiatrists, an interpretation further reinforced by evidence that more than half of patients had not consulted a physician for MH reasons in the year previous to an ER visit. In Quebec, 45% of PMI have no family physician [87], as opposed to 25% of the general population [88]. Previous research also reported that only 45% of PMI in Canada consulted their regular physician for MH problems [89]. Moreover, according to international studies, two thirds of PMI avoided seeking help altogether [22, 90], perhaps viewing their problems as too complex or specialized for GPs. Similarly, studies underline difficulties among GPs around providing MH care [91–93].

Regarding continuity of care, we found that most patients did not receive follow-up appointments within 7 to 30 days after an ER visit, or hospitalization for MH reasons. The situation had deteriorated in 2014–15 compared with 2005–05 for outpatient consultations after ER visits, but follow-up to hospitalization had improved considerably. Most studies identified barriers to care continuity among PMI [94], with average failure rates at 58% (range 18 to 67%) on outpatient follow-up after ER visits or hospitalizations [94–97], which coincides with our results. Improvement in outpatient consultation rates after hospitalizations may be explained by the MH reform priority on reducing hospitalizations [21], an international trend [7, 9, 98]. One-third of patients made return ER visits within 30 days, suggesting the persistence of problems in continuity of care. ER readmission rates for MI (with or without hospitalization) within 30 days range from 10 to 40% in the literature, depending on patient population [99, 100]. Severe MI (e.g. schizophrenia), co-occurring MI/SUD or MI/chronic physical illness are difficult to treat during an ER visit, particularly a first visit, requiring several medical consultations [34, 37, 52]. Our results showed that physical illness was the main reason for return to ERs among PMI. Medication use may also involve physical side effects requiring outpatient appointments proximal to discharge [1, 30].

Regarding appropriateness of care, 17% of patients were high/very high ER users, and often hospitalized. A systematic review found that high/very high ER users ranged from 0.03 to 18% among PMI [100], who were also high users of other services [34]; they tended to consult hospital-based professionals during crises [66]. High ER use may be explained by significant vulnerability, socioeconomic precariousness, and social isolation among PMI [72, 73], as well as the high prevalence of psychosocial problems associated with co-occurring physical conditions requiring specialized care combined with close follow-up in primary care [49]. Poor coordination of outpatient services and low overall continuity of care [37] frequently contribute to high hospitalization rates. Again, difficulties in the implementation of continuous services in the Quebec reform context may explain these results [10].

While most PMI made a single visit to an ER throughout the 2005–06 to 2014–15 period, nearly one in ten did so over three to five consecutive years during the same period. Moreover, ER use increased over three consecutive years, which may be explained by the chronicity often associated with MI. International studies reported that chronic episodic depression may last up to 3 years, especially when accompanied by suicidal ideation [101, 102]. Having two or more MI, SUD or severe MI such as personality disorders may also increase recurrent yearly ER use [43, 103]. Thus, our results highlight some of the challenges inherent in managing patients with complex MH profiles who require close monitoring or frequent medication readjustment by a multidisciplinary team in order to avoid recurring yearly ER use [10, 43]. The fact that recurring yearly ER use decreased over 5 consecutive years after 2005–06 suggests some post-reform improvement in the management of patients with serious and chronic problems. In Quebec, intensive case management and assertive community treatment targeting high ER users with serious MIs have been consolidated since the 2005–06 MH reform [21].

Even though only 3% of PMI were hospitalized for over 30 days, decreasing over time, their hospitalization rates were ten times that of patients without MI, while hospitalizations for this group were five times lower. An American study found that PMI remained in hospital 38% longer than others [48], while another reported hospitalization rates exceeding 30 days for PMI with severe disorders [59], similar to our results. Illness severity remains the main predictor for length of hospitalization, particularly in schizophrenia and psychosis [76, 83].

This study has limitations that should be acknowledged. First, outcomes of the reform may have been affected by the particular indicators selected to measure access, continuity and appropriateness of care. As well, the QICDSS used administrative databanks not originally designed for epidemiological research; they did not include statistics for physicians working in CLSCs who also follow vulnerable PMI [104]. Results from the QICDSS were also slightly underestimated due to a few percentages of missing data on ER diagnoses for the MH population. However, data extracted from the QICDSS may provide a basis for, and complement to, future epidemiological research. Finally, as this study involved ER service use in the Quebec MH population, comparative studies are needed for other Canadian provinces or countries to more adequately evaluate the quality of MHS.

## Conclusions

This study is the first to evaluate recent data on MHS quality indicators related to ER use for a cohort of PMI in Quebec, and to compare results with data for the period coinciding with implementation of the Quebec MH reform (2005–06). While we hypothesized that the reform would improve access, continuity, and appropriateness of care, and reduce ER attendance, results did not strongly support these hypotheses. Some improvement occurred over the 10-year period, yet gains among PMI were generally smaller than among patients without MI. In fact, while slightly fewer PMI visited ERs over time, the decrease among patients without MI was twice as great. With service improvement targets unmet, MH seems to remain the “Cinderella” of the Quebec health and social service system. The literature on MHS quality improvement supports various strategies which, if better implemented, could be integrated into the Quebec system to increase ER functioning and enhance coordination between ERs and MH community networks. These include the consolidation of MH and primary care services and follow-up practices such as assertive community treatment (ACT), intensive community management (ICM) or case management, targeting patients with more severe or co-occurring MH profiles, including high or very high ER users. Shared-care, involving closer collaboration between psychiatrists and other MH primary care providers, particularly GPs, may also favor MHS quality improvement. More systematic and routine outpatient follow-up to ER visits or hospitalizations, based on inter-organizational collaboration, may also reduce return ER visits within 30 days. Improving access and continuity of care for the most vulnerable individuals may also reduce the high prevalence of ER use in this population. Alternative services such as crisis resolution teams and home intervention teams could also be implemented with good effect. Overall, there is much room for quality improvement in MHS, including better support for ERs by integrating them within solid community-based MH networks.

## Abbreviations

ACT: Assertive community treatment
AD/HD: Attention deficit disorder with or without hyperactivity
CLSC: Community health and social service center
DNS: Data not shown
ER: Emergency room
GP: General practitioner
HIN: Health insurance number
ICD: International Classification of Diseases
ICM: Intensive case management
INSPQ: Quebec National Institute of Public Health
MED-ECHO: Hospitalization databank/Maintenance et exploitation des données pour l’étude de la clientèle hospitalière
MHS: Mental health services
MI: Mental illnesses
PMI: Patients with mental illnesses
QICDSS: Quebec Integrated Chronic Disease Surveillance System
RAMQ: Quebec Health Insurance Regime
SUD: Substance use disorders
